# Transition from drug lag to drug loss in pediatric oncology: a comprehensive evaluation of the Japan-U.S. approval gap

**DOI:** 10.3389/fphar.2026.1882440

**Published:** 2026-08-26

**Authors:** Kana Yamada, Hiroyuki Sato, Akihiro Hirakawa

**Affiliations:** Department of Clinical Biostatistics, Graduate School of Medical and Dental Sciences, Institute of Science Tokyo, Tokyo, Japan

**Keywords:** drug lag, drug loss, pediatric global development strategy, pediatric oncology, RACE act, regulatory science

## Abstract

**Introduction:**

Pediatric drug development in Japan faces significant challenges caused by the case rarity and limited commercial incentives. Although the United States (U.S.) RACE Act in 2017 has accelerated pediatric oncology approvals in the U.S., it has coincided with a widening approval gap between the U.S. and Japan. This retrospective observational study aimed to clarify the current status of pediatric oncology drug lag and apparent drug loss through 2025 and to explore structural factors associated with pediatric approval status in Japan.

**Methods:**

We assessed the Japanese approval status of pediatric oncology drugs with U.S. Food and Drug Administration indications from 2000 to 2025. Drug lag was evaluated using Kaplan-Meier analysis, primarily focusing on drugs that successfully obtained Japanese approval, and clinical development factors associated with apparent drug loss were analyzed using Fisher’s exact test and multivariable logistic regression. Decision tree analysis (CART) was performed to identify the structural hierarchy of clinical characteristics associated with Japanese approval status.

**Results:**

The approval rate of pediatric drug-indication pairs in Japan significantly declined from 81.8% before 2017 to 31.8% after 2017. Kaplan-Meier analysis suggested that, among drug-indication pairs that ultimately achieved pediatric approval in Japan, the median time to approval was shortened from 48.9 months to 21.1 months. Multivariate and CART analysis identified that the trial phase supporting approval in the U.S. and sponsor type were the key factors most strongly associated with approval in Japan. When the U.S. study supporting approval was limited to early-phase trials or was absent, 92.9% of drugs failed to achieve approval in Japan.

**Discussion:**

While delays in drug approval may have shortened, the disparity in therapeutic access between Japan and the U.S. is increasingly driven by persistent not yet approved in Japan. To address this gap, Japan should transition toward an integrated strategy that balances robust domestic bridging—leveraging foreign pediatric and Japanese adult data extrapolation—with proactive participation in the global clinical development engine from its earliest phases.

## 1 Introduction

Pediatric oncology drug development remains a major challenge because pediatric cancers are rare and biologically heterogeneous diseases, resulting in limited patient populations for clinical trials and reduced commercial incentives for drug development (Adamson et al., 2014). Many pediatric diseases associated with unapproved drugs in Japan are rare diseases, and oncology has been reported as one of the major therapeutic areas with persistent unmet medical needs (Haigo et al., 2025). In addition, the implementation rate of pediatric clinical trials for anticancer agents is lower than that in other therapeutic areas, which further contributes to delayed access to innovative therapies for pediatric patients in Japan (The Office of Pharmaceutical Industry Research, 2021).

In the U.S., pediatric oncology development has accelerated through both scientific advances and regulatory reforms. A major milestone was the enactment of the Research to Accelerate Cures and Equity (RACE) for Children Act in 2017 and its implementation in 2020. The RACE Act requires sponsors to evaluate new adult oncology drugs for pediatric development when the molecular target is relevant to pediatric cancers, even for orphan-designated products that had previously been exempt from mandatory pediatric studies (U.S. Congress, 2017). Following implementation of the RACE Act, pediatric clinical trials in oncology have reportedly been initiated earlier, and pediatric oncology approvals in the U.S. have increased substantially (Liu and Kesselheim, 2024; U.S. Food and Drug Administration, 2025).

Previous Japanese policy reports have suggested that the number of pediatric oncology approvals in the U.S. increased rapidly between 2017 and 2023, resulting in a widening approval gap between the U.S. and Japan (The Office of Pharmaceutical Industry Research, 2023). However, whether this expanding gap is mainly attributable to approval delay (drug lag), failure to obtain approval (apparent drug loss), or both remains unclear. Previous studies have shown that pediatric drug lag in Japan has gradually improved, particularly when global development strategies are adopted (Ueyama et al., 2021). Therefore, the recent widening of the approval gap may reflect not only conventional approval delay but also structural apparent drug loss, in which products approved overseas fail to reach Japanese pediatric patients.

In Japan, pediatric oncology approvals are often supported by overseas clinical trial data or adult data, rather than by domestic pediatric development (Haigo et al., 2025). This reflects the fact that pediatric oncology clinical trials are rarely conducted solely within Japan; therefore, participation in multinational clinical trials (MRCT) is considered important for achieving timely pediatric drug approval (The Office of Pharmaceutical Industry Research, 2022). These observations suggest that development strategy, including sponsor type, study phase supporting approval in the U.S., presence/absence of participation in global development programs, and the use of extrapolation from adult data, may influence pediatric approval status in Japan. However, the structural relationship among these factors has not been sufficiently investigated.

Although previous studies have described Japanese approval trends and regulatory policy changes in the oncology field (The Office of Pharmaceutical Industry Research, 2021; The Office of Pharmaceutical Industry Research, 2022; The Office of Pharmaceutical Industry Research, 2023), few have comprehensively evaluated whether the expanding pediatric approval gap after 2017 is primarily caused by drug lag or apparent drug loss. Therefore, this study aimed to clarify the current status of pediatric oncology drug lag and apparent drug loss between the U.S. and Japan by extending previous analyses through 2025 using FDA Pediatric Oncology Drug Approvals data (U.S. Food and Drug Administration, 2025). First, we evaluated whether the expanding approval gap after 2017 was mainly attributable to drug lag or apparent drug loss. Subsequently, we analyzed the association between U.S. pediatric development characteristics and Japanese pediatric approval status. Finally, we explored the structural relationship among these factors to better understand patterns associated with pediatric approval status in Japan. Based on these findings, we discuss potential regulatory and development strategies to reduce pediatric oncology apparent drug loss and improve access to innovative therapies for Japanese pediatric patients.

## 2 Materials and methods

### 2.1 Data source and extraction of drugs with pediatric indications

This retrospective observational study was conducted and reported in accordance with the Strengthening the Reporting of Observational Studies in Epidemiology (STROBE) guidelines. The completed STROBE reporting checklist is provided in the Supplementary Material (Supplementary Material: STROBE Checklist).

This study used the U.S. Food and Drug Administration (FDA) Pediatric Oncology Drug Approvals database as the primary data source to identify anticancer drugs that obtained pediatric indications in the U.S. between January 2000 and December 2025 (U.S. Food and Drug Administration, 2025) and extracted drugs. When a drug received multiple pediatric approvals for different age groups (e.g., initial approval for patients aged ≥ 12 years followed by expanded approval for patients aged ≥ 1 year), only the earliest pediatric approval date was used for analysis.

For each drug, Japanese pediatric approval status was assessed by reviewing the Japanese package insert, specifically the “Indications and Usage” and “Dosage and Administration” sections (Pharmaceuticals and Medical Devices Agency, 2026). Drugs were considered to have pediatric approval in Japan if the approved indication was consistent with that in the U.S. and the Japanese labeling did not explicitly limit use to adult patients only. Drugs were categorized into three groups: (1) pediatric approval obtained in Japan, (2) approved only for adults in Japan without pediatric indication, and (3) not approved in Japan.

### 2.2 Variable definitions and classification

Apparent drug loss was defined as pediatric oncology drug-indication pairs approved by the U.S. FDA that were “not yet approved in Japan” as of the data cutoff date (31 December 2025). Drug-indication pairs without Japanese approval were conveniently classified as apparent drug loss regardless of ongoing clinical development; 10 such pairs were under development in Japan at the data cutoff date. The following characteristics of U.S. pediatric clinical development that supported FDA approval were extracted and evaluated as the candidate explanatory variables for Japanese pediatric approval status. These variables were selected to assess how development strategy and clinical trial design were associated with drug lag and/or apparent drug loss between the U.S. and Japan. Regulatory and clinical development information was collected from multiple public sources, including Drugs@FDA, FDA Prescribing Information (USPI), Multi-Discipline Review documents, ClinicalTrials.gov, and publicly available sponsor information (U.S. Food and Drug Administration, 2026a; U.S. National Library of Medicine, 2026).

FDA Approval Pattern: The timing of pediatric approval relative to adult approval was classified into three categories: (1) same timing (simultaneous adult and pediatric approval), (2) adult followed by pediatric approval, and (3) pediatric-first or pediatric-only approval.

Sponsor Type: Sponsors were classified as Mega Pharma or non-Mega Pharma. Following established industry benchmarks, Mega Pharma was defined as companies with annual global sales exceeding USD 5 billion.

U.S. Study Phase: The latest pediatric clinical study phase contributing to U.S. approval was classified into three categories: (1) Phase II, (2) Phase II/III or Phase III, and (3) others, including Phase I only or no dedicated pediatric study.

Multi-Regional Clinical Trial (MRCT) Participation: Clinical studies were classified into two categories: MRCT and non-MRCT (e.g., U.S. domestic trials).

Primary Endpoint: The primary endpoint of the latest pediatric study in U.S. was categorized into three groups: (1) time-to-event endpoints (including event-free survival [EFS], relapse-free survival [RFS], disease-free survival [DFS], progression-free survival [PFS] or Overall Survival [OS]), (2) response rate, and (3) other endpoints (including pharmacokinetics [PK], safety, or cases without pediatric efficacy data).

Pediatric Inclusion in Adult Trial: Whether the Ages Eligible for Study field in ClinicalTrials.gov included both adult ( ≥ 18 years) and pediatric ( <18 years) patients (Yes or No).

Tumor Type: Diseases were classified according to the primary site and histology into hematologic or solid tumor.

Extrapolation from Adult Data: Whether the U.S. pediatric approval relied on the extrapolation of efficacy and safety data from adult clinical trials (Yes or No).

## 3 Statistical analysis and analytical workflow

This study consisted of four sequential analyses designed to evaluate pediatric oncology drug lag and apparent drug loss between the U.S. and Japan, as well as to identify clinical development factors in the U.S. associated with these approval gaps.

First, to characterize the current landscape of the Japan–U.S. pediatric approval gap, annual trends in pediatric oncology approvals in the U.S. and their corresponding Japanese approval status were evaluated from 2000 to 2025. The cumulative number of pediatric approvals in the U.S. and their approval status in Japan were visualized using stacked bar graphs. In addition, the proportion of Japanese pediatric approvals among U.S.-approved pediatric oncology drugs before and after 2017 was compared using frequency analysis and Fisher’s exact test to assess the potential impact of the RACE Act. Second, to determine whether the expanding approval gap was primarily attributable to approval delays (drug lag) or to the status of being “not yet approved in Japan” (apparent drug loss), we first evaluated the status of drug lag. This analysis included drug-indication pairs approved in both countries, as well as those currently under development in Japan ( n=10 pairs), which were treated as censored at the data cutoff date (31 December 2025). The time interval between U.S. and Japanese pediatric approvals was calculated using the U.S. and Japanese approval date as the starting point and event date, respectively. For drugs approved earlier in Japan than in the U.S., Day 0 was treated as a censored observation. Kaplan–Meier estimation was performed to evaluate time-to-approval and compare drug lag before and after 2017. The year 2017 was selected as the cutoff based on previous reports demonstrating a marked increase in U.S. pediatric oncology approvals and an expanding approval gap between Japan and the U.S. around this period (The Office of Pharmaceutical Industry Research, 2023), as well as its coincidence with the enactment of the RACE Act. A sensitivity analysis was also performed using 2020, the year of its implementation, as an alternative threshold. Third, to identify clinical development factors associated with apparent drug loss, drugs approved in the U.S. through 2023 were analyzed. Drugs approved in 2024–2025 were excluded from this analysis because they may still obtain pediatric approval in Japan within the observed upper limit of median lag period and therefore could represent temporary drug lag rather than definitive apparent drug loss. Among drug-indication pairs approved in the U.S. through 2023, 6 remained under clinical development in Japan at the data cutoff date and, although potentially representing ongoing drug lag, were classified as apparent drug losses according to the study definition. Associations between Japanese pediatric approval status and U.S. development characteristics were evaluated using frequency analysis and Fisher’s exact test. Furthermore, multivariable Firth logistic regression analysis using a backward variable selection was performed to identify predictors of Japanese pediatric approval status and to calculate adjusted odds ratios (OR) with 95% confidence intervals (CI). Multicollinearity was assessed using Spearman’s rank correlation coefficients, and the primary endpoint of the latest pediatric study in U.S. was excluded from the multivariable model due to its high correlation with U.S. study phase supporting approval (ρ≥0.6) . Given the limited sample size relative to the number of predictors, this multivariable model was positioned as an exploratory analysis. Finally, to clarify the structural and hierarchical relationships among factors associated with pediatric approval status in Japan, decision tree analysis using the Classification and Regression Tree (CART) method was performed. This analysis aimed to identify combinations of development characteristics associated with approval success or failure, providing a structural understanding of the pathways leading to apparent drug loss in Japan. To broadly capture the overall landscape of factors contributing to apparent drug loss, the initial tree was constructed with the complexity parameter (CP) set to 0.01 and the minimum number of observations required to attempt a split (min-split) set to 5. Given the limited sample size (N = 62) and the inherent risk of overfitting with small terminal nodes, this CART analysis was positioned as exploratory. To mitigate these risks, 10-fold cross-validation was conducted to estimate the cross-validation error and identify the optimal tree complexity. Subsequently, post-pruning was applied based on the minimum cross-validation error (min-error) rule to derive an optimized final model. Furthermore, the structural stability of the selected variables was verified by calculating their selection frequencies across 1,000 bootstrap iterations. All statistical analyses were performed using R software (Version 4.5.2).

## 4 Results

### 4.1 Overview of pediatric oncology drug approvals and Japanese approval status

A total of 77 pediatric oncology drug-indication pairs approved by the U.S. Food and Drug Administration (FDA) between 2000 and 2025 were identified using the FDA Pediatric Oncology Drug Approvals database (Supplementary Table S1).

**FIGURE 1:**
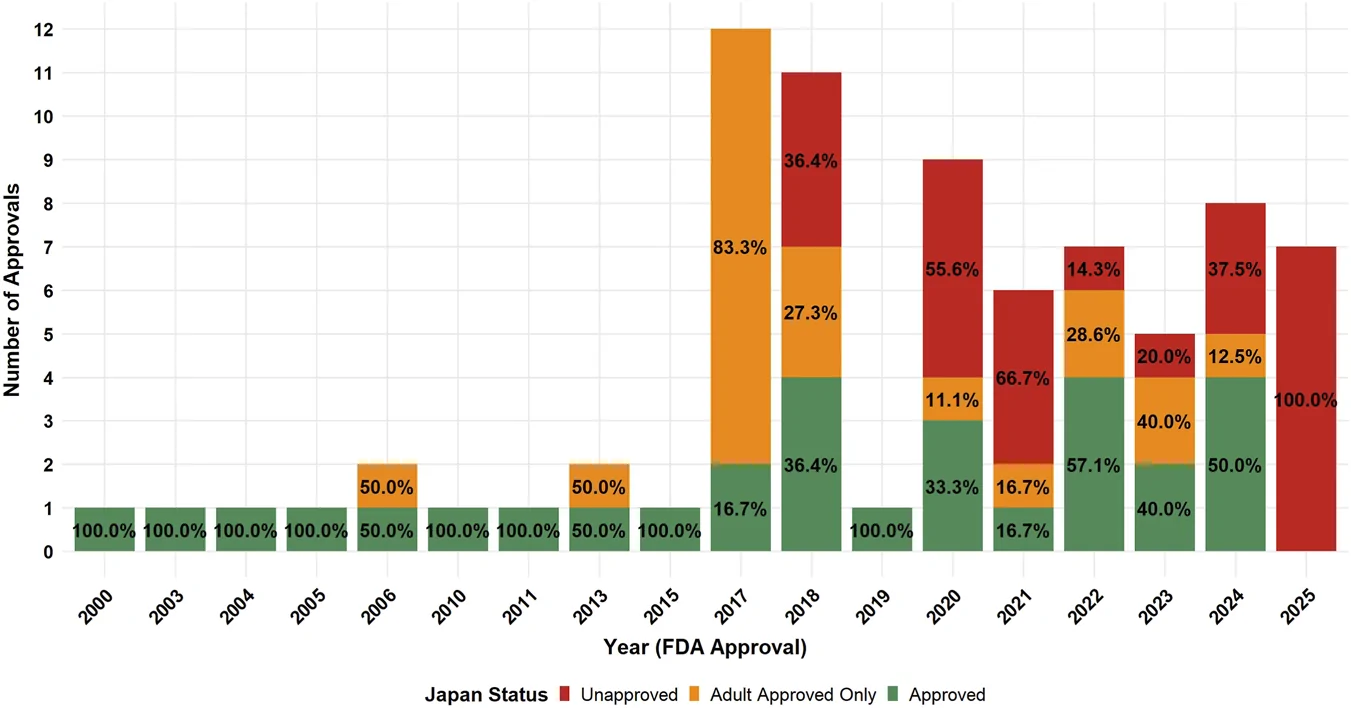
Annual trends in U.S. pediatric oncology drug approvals (2000–2025) and corresponding Japanese approval status. Green indicates pediatric approval in Japan, orange indicates adult approval only, and red indicates no approval in Japan.

Pediatric oncology approvals in the U.S. continued to increase after 2017, whereas a substantial proportion of these drug-indication pairs had not obtained pediatric approval in Japan (Figure 1). Of the 77 total pediatric oncology drug-indication pairs, 30 (39.0%) were approved for pediatric use in Japan, 22 (28.6%) were approved only for adult indications, and 25 (32.5%) remained completely unapproved in Japan. The approval gap became particularly prominent after 2017. Before 2017, 9 of 11 drug-indication pairs (81.8%) had obtained pediatric approval in Japan, whereas only 21 of 66 drug-indication pairs (31.8%) approved after 2017 were subsequently approved for pediatric use in Japan. Conversely, the proportion of drug-indication pairs without Japanese pediatric approval increased from 18.2% before 2017 to 68.2% after 2017, representing a statistically significant decline in Japanese pediatric approval rates after 2017 (p=0.0025) (Table 1). Our extended analysis through 2025 confirmed that this gap has persisted despite a gradual increase in pediatric approvals in Japan, suggesting ongoing drug lag and/or apparent drug loss in pediatric oncology drug development. To clarify whether this expanding gap was primarily driven by drug lag or apparent drug loss, we first focused on drug lag, as previous studies have suggested that pediatric drug lag in Japan has been gradually improving, particularly when global development strategies are adopted (Ueyama et al., 2021). Therefore, we evaluated the time to Japanese pediatric approval by comparing approval lag before and after 2017.

**TABLE 1:** Comparison of Japanese pediatric approval rates before and after 2017.

Era (Threshold 2017)	Total (N)	Approved in Japan, n (%)	Not approved in Japan, n (%)
Pre-2017	11	9 (81.8)	2 (18.2)
Post-2017	66	21 (31.8)	45 (68.2)

Statistical test: Fisher’s exact test, p=0.0025 .

### 4.2 Drug lag before and after 2017

**FIGURE 2:**
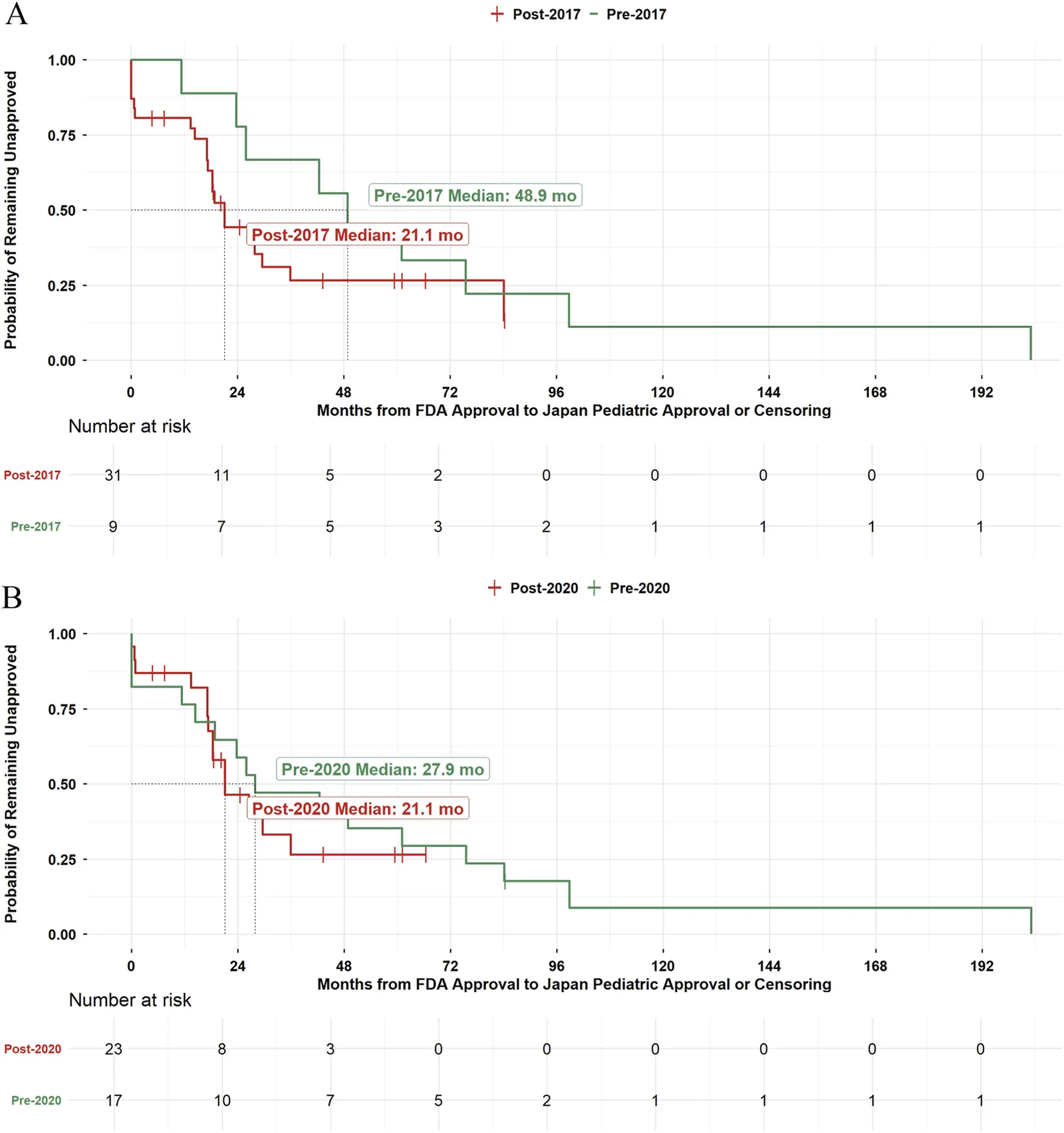
**(A)** Kaplan-Meier analysis of time from U.S. pediatric approval to Japanese pediatric approval before and after 2017. **(B)** Kaplan-Meier analysis of time from U.S. pediatric approval to Japanese pediatric approval before and after 2020.

To clarify whether the expanding approval gap observed after 2017 was primarily attributable to drug lag, we compared the time from U.S. pediatric approval to Japanese pediatric approval between drugs approved before and after 2017 using Kaplan-Meier analysis. The median approval lag was shorter for drugs approved after 2017 than for those approved before 2017 among drug-indication pairs that ultimately received pediatric approval in Japan. The median time to Japanese pediatric approval was 21.1 months in the post-2017 group, compared with 48.9 months in the pre-2017 group (Figure 2A). In the post-2017 group, most drugs that eventually obtained pediatric approval in Japan were approved within approximately 2 years after U.S. approval, whereas approvals in the pre-2017 group required substantially longer periods. These findings suggest that, among drug-indication pairs that ultimately achieved pediatric approval in Japan, the time to approval has shortened over time. Additionally, a sensitivity analysis using 2020 as the cutoff year yielded results similar to those observed with the 2017 threshold (Figure 2B). Furthermore, a supplementary Kaplan-Meier analysis including all U.S.-approved drug-indication pairs showed that the curve for the post-2017 cohort reached a plateau after approximately 24 months. This suggests that a substantial proportion of drugs remain unapproved in Japan beyond this period, potentially reflecting a broader overall access gap associated with apparent drug loss (Supplementary Figure S1).

### 4.3 Factors associated with apparent drug loss in Japan

To identify the clinical development characteristics associated with apparent drug loss in Japan, we analyzed a subset of 62 drug-indication pairs that obtained pediatric approval in the U.S. through 2023.

Univariate analysis using Fisher’s exact test showed that certain clinical development characteristics were associated with Japanese approval status (Table 2). Specifically, higher Japanese approval rates were observed when the study supporting U.S. approval was Phase II or later (p=0.0045) and when response rate was used as the primary efficacy endpoint (p=0.0025) . Conversely, the use of extrapolation from adult data for U.S. approval was associated with a lower Japanese approval rate compared with developments that did not rely on extrapolation (31.2% vs. 53.3%, p=0.1219 ). MRCT Participation was associated with a higher approval rate in Japan compared with non-MRCT developments (53.3% vs. 31.2%, p=0.1219 ). To further explore these distributions, a supplementary Fisher’s exact test was conducted by stratifying the unapproved category into “approved only for adults in Japan without pediatric indication” and “not approved in Japan” (Supplementary Table S2).

**TABLE 2:** Univariate analysis of U.S. clinical development characteristics associated with Japanese pediatric approval status.

Variable	Category	Total (N)	Approved in Japan		p -value
			Yes, n (%)	No, n (%)	
MRCT participation	Non-MRCT	32	10 (31.2)	22 (68.8)	0.1219
	MRCT	30	16 (53.3)	14 (46.7)	​
U.S. study phase	Phase II	38	21 (55.3)	17 (44.7)	0.0045
	Phase III or II/III	10	4 (40.0)	6 (60.0)	​
	Others^a^	14	1 (7.1)	13 (92.9)	​
Extrapolation from adult data	No	30	16 (53.3)	14 (46.7)	0.1219
	Yes	32	10 (31.2)	22 (68.8)	​
Tumor type	Hematologic	29	13 (44.8)	16 (55.2)	0.7974
	Solid	33	13 (39.4)	20 (60.6)	​
FDA approval pattern	Adult first	14	6 (42.9)	8 (57.1)	0.6292
	Same timing	37	14 (37.8)	23 (62.2)	​
	Pediatric only	11	6 (54.5)	5 (45.5)	​
Pediatric inclusion in adult trial	No	48	19 (39.6)	29 (60.4)	0.5475
	Yes	14	7 (50.0)	7 (50.0)	​
Sponsor type	Non-mega pharma	29	10 (34.5)	19 (65.5)	0.3098
	Mega pharma	33	16 (48.5)	17 (51.5)	​
Primary endpoint	EFS/RFS/DFS/PFS/OS	8	2 (25.0)	6 (75.0)	0.0025
	Response rate	31	20 (64.5)	11 (35.5)	​
	Others^b^	23	4 (17.4)	19 (82.6)	​

^a^ Others includes Phase I only or no dedicated pediatric efficacy study.

^b^ Others includes pharmacokinetics, safety, or no formal pediatric efficacy endpoint.

In the final model derived from multivariable Firth logistic regression with backward elimination, the U.S. study phase supporting approval was the factor most strongly associated with Japanese pediatric approval (Table 3). In this final model, when the study supporting U.S. approval was Phase II, the odds of achieving Japanese pediatric approval increased 12.11-fold ( OR=12.11 , 95% CI: 2.48–120.94, p=0.001 ). Phase III or II/III developments also showed a significantly higher odds ratio ( OR=9.09 , 95% CI: 1.20–116.06, p=0.032 ). Sponsor type was the only other variable retained after the selection process, though its independent association was not statistically significant at the 0.05 level ( OR=2.20 , 95% CI: 0.71–7.14, p=0.171 ). Other candidate factors, such as extrapolation from adult data and MRCT participation, were eliminated during the backward selection. Although the multivariable model suggested that the U.S. study phase supporting approval and sponsor type were the factors most strongly associated with Japanese pediatric approval, the elimination of other clinical development factors during the variable selection process suggests that their effects may be interdependent and governed by a complex hierarchical structure. To further explore these interactions and identify factors associated with apparent drug loss, decision tree analysis was subsequently performed to map the structural relationships among these factors.

**TABLE 3:** Multivariable Firth logistic regression analysis of clinical development factors associated with Japanese pediatric approval (Final model after backward elimination).

Variable and category	Odds ratio	95% CI^a^	p -value
U.S. Study phase: Phase II^b^	12.11	2.48–120.94	0.001
U.S. Study phase: Phase III or II/III^b^	9.09	1.20–116.06	0.032
Sponsor type: Mega pharma (Yes)	2.20	0.71–7.14	0.171

^a^ Confidence intervals derived from Firth logistic regression.

^b^ Reference category: Others.

The final model was selected using a backward elimination method with a significance level for retention of 0.20.

### 4.4 Structural hierarchy of factors associated with Japanese pediatric approval

To clarify the structural hierarchy and combinations of clinical development characteristics associated with pediatric approval outcomes in Japan, a decision tree analysis using the CART method was performed.

**FIGURE 3:**
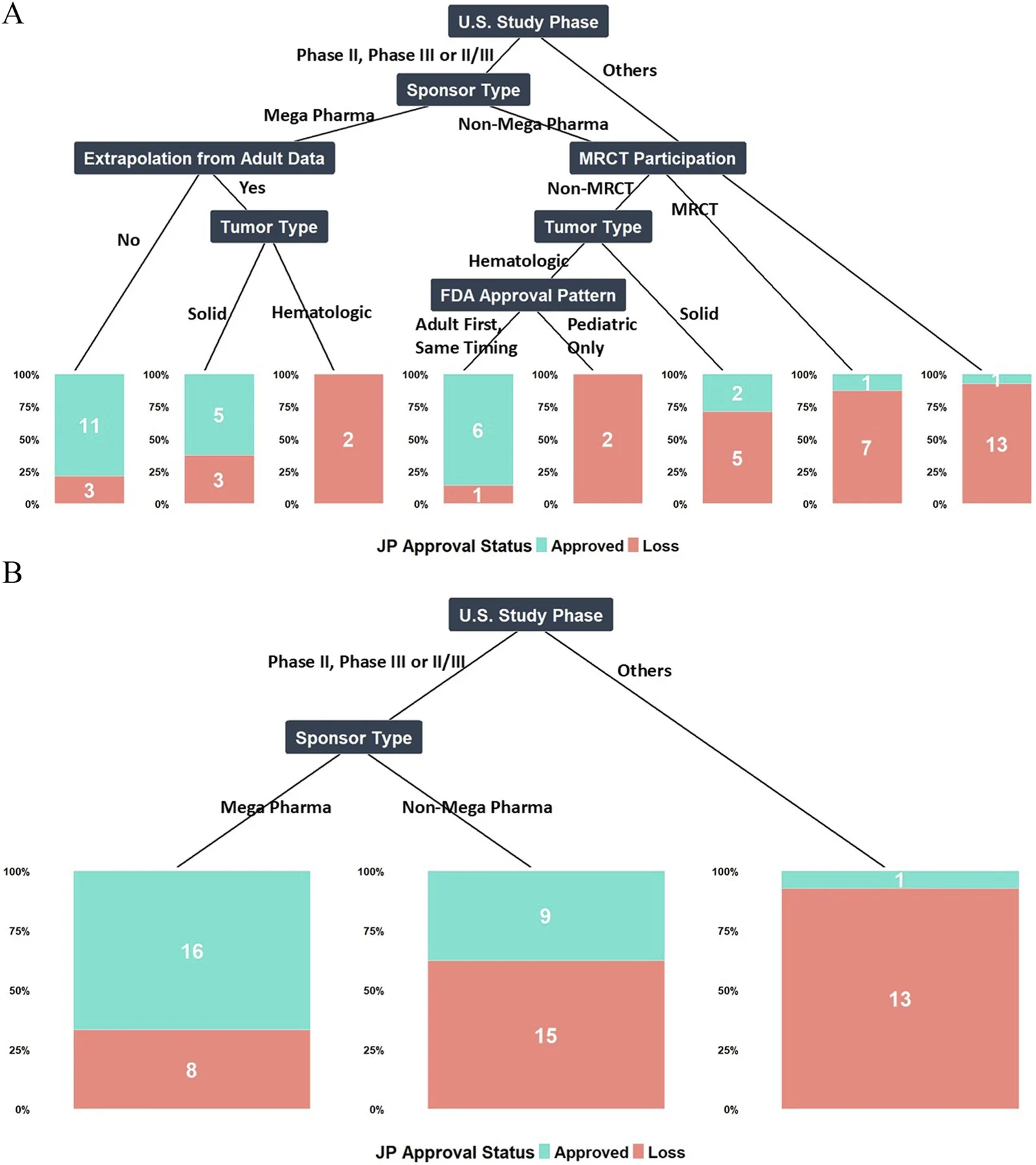
**(A)** Decision tree analysis of factors associated with Japanese pediatric approval (Initial full model). **(B)** Decision tree analysis of factors associated with Japanese pediatric approval (Optimized post-pruned model).

To broadly explore the potential pathways leading to apparent drug loss, an unpruned initial tree was constructed using predefined stopping criteria (Figure 3A). This analysis suggested the U.S. study phase supporting approval was the factor most strongly associated with Japanese pediatric approval, followed by sponsor type, extrapolation from adult data, and MRCT participation. For products that reached Phase II or later in the U.S., outcomes were further differentiated by sponsor type. Among Phase II/III developments conducted by Mega Pharma sponsors, the highest success rate was observed in the group that did not rely on extrapolation from adult data, where 78.6% (11/14) of drug-indication pairs were successfully approved. Conversely, when Mega Pharma sponsors utilized an extrapolation approach, the Japanese approval rate dropped to 50.0% (5/10). Among drug-indication pairs developed by non-Mega Pharma sponsors, MRCT participation was significantly associated with apparent drug loss; a remarkably high rate of apparent drug loss (87.5%, 7/8) was observed when MRCT was utilized, primarily due to the exclusion of Japan from these global frameworks.

Given the small sample size, the deeply split initial tree was subject to a high risk of overfitting. To assess model validity and identify the optimal tree complexity, 10-fold cross-validation was conducted (Table 4). The cross-validation error reached its minimum at a tree size of two splits (Complexity Parameter [CP] = 0.058, cross-validation error = 0.885). Based on the min-error rule, post-pruning was applied to derive the optimized final model (Figure 3B). This pruned tree identified the U.S. study phase supporting approval as the primary splitting variable, followed by sponsor type among drug-indication pairs with a U.S. study phase reaching Phase II or later. Developments by Mega Pharma sponsors achieved an overall approval rate of 66.7% (16/24), whereas those by non-Mega Pharma sponsors had an approval rate of only 37.5% (9/24). Finally, the stability of the selected variables was assessed by bootstrap analysis with 1,000 iterations. As shown in the selection frequency results (Table 5), the U.S. study phase supporting approval and sponsor type were consistently selected as primary splitting variables in 83.2% and 65.8% of the bootstrap samples, respectively. Other factors, such as MRCT participation (39.6%) and tumor type (39.4%), demonstrated moderate selection frequencies, while extrapolation from adult data (24.4%), pediatric inclusion in adult trials (16.6%), and FDA approval pattern (13.4%) were substantially less stable. These validation results suggest that the U.S. study phase supporting approval and sponsor type were the factors most consistently associated with Japanese pediatric approval status, despite the limited sample size.

**TABLE 4:** Cross-validation metrics for decision tree optimization.

Complexity parameter (CP)	Number of splits	Relative training error	CV error	Standard error (SE)
0.154	0	1.000	1.000	0.149
0.058	2	0.692	0.885	0.146
0.038	5	0.500	0.962	0.149
0.010	7	0.423	0.923	0.148

**TABLE 5:** Variable selection frequency across 1,000 bootstrap iterations.

Variable	Frequency	Percent (%)
U.S. Study phase	832	83.2
Sponsor type	658	65.8
MRCT participation	396	39.6
Tumor type	394	39.4
Extrapolation from adult data	244	24.4
Pediatric inclusion in adult trials	166	16.6
FDA approval pattern	134	13.4
No split (root only)	5	0.5

## 5 Discussion

The development of pediatric oncology therapeutics has reached a critical turning point characterized by an expanding gap in access to treatments between the U.S. and Japan. Our study suggests that the RACE for Children Act may have contributed to an increase in pediatric drug approvals in the U.S.; however, the approval gap between the two countries continues to widen.

Traditionally, regulatory discussions in Japan have focused on “drug lag” defined as delays in domestic approval compared with global launches (Maeda et al., 2023; Inoue et al., 2025). However, our analysis demonstrated that the median time from U.S. pediatric approval to Japanese pediatric approval decreased substantially from 48.9 months before 2017 to 21.1 months after 2017. These findings suggest that, among drug-indication pairs that ultimately achieved pediatric approval in Japan, the time to approval appeared shorter in the later period. Instead, the central challenge is increasingly characterized by “apparent drug loss,” which manifests as persistent non-approval in Japan.

Frequency analysis and Fisher’s exact test demonstrated that drugs with response rate as the primary endpoint in U.S. submissions were more likely to achieve approval in Japan, whereas those evaluated based on safety, pharmacokinetics (PK), or time-to-event (TTE) endpoints were less likely to be approved. Among drug-indication pairs evaluated primarily for safety, PK, or without pediatric development in the U.S., 19 failed to achieve approval in Japan, of which 12 lacked any pediatric evaluation. The absence of pediatric data in U.S. development likely makes it difficult to conduct clinical studies in Japan, thereby discouraging the initiation of pediatric development programs. For drug-indication pairs requiring TTE endpoints, the longer evaluation period and larger sample size requirements pose substantial challenges for conducting independent domestic trials in Japan. In our dataset, the median sample size was 36 patients for response rate-based studies, compared with 139 patients for those using TTE endpoints. Consistent with these findings, among programs limited to Phase I trials or lacking pediatric development, 13 of 14 failed to achieve approval in Japan, despite most of these drug-indication pairs already being approved for adult indications in Japan. Additionally, 11 of the 13 drug-indication pairs that were not approved in Japan were intended for biomarker-defined populations or ultra-orphan diseases, for which dedicated pediatric clinical trials are often difficult to conduct because of the extremely limited patient populations. Only 2 of these drug-indication pairs were supported by pediatric studies in the U.S., suggesting that opportunities for early Japanese participation in global pediatric development were inherently limited. Another factor increasing the likelihood of non-approval in Japan is when U.S. trial relies on an adult data extrapolation approach. Approximately half of pediatric drug-indication pairs in the U.S. are approved based on extrapolation from adult data. Although this approach accelerates access in the U.S., it creates a critical evidence gap for Japan, as pediatric-specific data required for bridging are not generated when global pediatric trials are omitted.

Multivariable logistic regression analysis using a backward variable selection demonstrated that, after adjusting for other candidate clinical development factors, the U.S. study phase supporting approval and sponsor type were the factors most strongly associated with Japanese pediatric approval. In contrast, factors such as extrapolation from adult data and MRCT participation showed attenuated associations in the multivariate model, suggesting that these factors themselves may not independently influence pediatric approval status but instead may be associated with other development characteristics. Therefore, regression analysis alone may not fully capture the pathways leading to drug development failure, highlighting the value of CART analysis. CART analysis generated several critical hypotheses regarding the structural pathways leading to apparent drug loss.

First, programs limited to Phase I or lacking dedicated pediatric efficacy studies were associated with a high apparent drug loss rate (92.9%). This finding suggests that the absence of such pediatric evidence may rarely be overcome by downstream strategies. Second, the hierarchical structure of the pruned model generated the hypothesis that advancing to Phase II or later in the U.S. is not inherently sufficient for Japanese approval; rather, sponsor type acts as the important secondary bottleneck. While Mega Pharma programs achieved a 66.7% approval rate, non-Mega Pharma programs achieved only 37.5%. Many of these non-Mega Pharma programs were driven by U.S.-centric academic networks, such as the National Cancer Institute (NCI) or the Children’s Oncology Group (COG). Because these frameworks inherently lack a commercial sponsor, there is no corporate entity dedicated to driving regulatory submissions to Japanese authorities. This suggests that even when robust late-phase clinical data is obtained in the U.S., without proactive engagement and outreach from the Japanese side to these academic networks, such data may not readily translate into Japanese approval, ultimately resulting in apparent drug loss. In contrast, the unpruned tree implied that programs led by Mega Pharma, supported by Phase II or later studies without reliance on adult extrapolation, achieved a high approval rate in Japan (78.6%, 11/14). In all such cases, foreign response rate data were effectively utilized, suggesting efficient pathways to Japanese approval. This conditional branching generates the hypothesis that securing pediatric efficacy evidence to support U.S. approval, backed by the abundant resources of Mega Pharma, and actively leveraging this data for Japanese regulatory submissions constitutes the hierarchical pathway with the highest probability of approval. Finally, the unpruned tree suggested a counterintuitive trend regarding MRCT: when non-Mega Pharma programs utilized MRCT, they showed a high failure rate of 87.5%. In these cases, it was confirmed that Japan was actually excluded from the global trials. This suggests the hypothesis that resource, cost, or regulatory constraints disproportionately hinder smaller sponsors from including Japan in their global frameworks, ultimately leading to apparent drug loss.

To address apparent drug loss in pediatric oncology in Japan, strategic interventions by Japanese stakeholders at the early stages of U.S. development may be important. As the primary split in the CART analysis demonstrated, establishing late-phase efficacy data in the U.S. serves as an important prerequisite for obtaining approval in Japan. Therefore, proactive intervention by Japan in U.S. development during its early phases, before late-phase clinical trial designs are finalized, could be highly beneficial. Furthermore, by proactively collaborating with U.S. academic networks and smaller sponsors during these initial stages of clinical development, Japanese stakeholders might help mitigate the risk of Japan being structurally excluded from subsequent late-phase evidence generation. However, early participation in global trials is not always feasible, particularly for ultra-orphan diseases or biomarker-defined populations. In such settings, complementary approaches—such as rational extrapolation from foreign or adult data, or registry-based evidence generation—could play an essential role in supporting pediatric drug approvals.

To translate these strategic concepts into concrete actions and comprehensively address the structural barriers to pediatric drug access, a multi-faceted approach involving various stakeholders is essential. First, institutional frameworks could promote Japan’s early participation in global development, including mechanisms analogous to the RACE for Children Act that encourage pediatric evaluation based on molecular targets. Second, to operationalize proactive engagement with non-Mega Pharma programs—which are largely driven by U.S. academic networks—formal collaborative frameworks may be beneficial. Domestic networks such as the Japan Children’s Cancer Group (JCCG) could build strategic alliances or joint working groups with the COG and the NCI to facilitate early discussions on integrating Japan into global protocols. Furthermore, because these U.S. academic frameworks often lack corporate sponsors, partnerships with Japanese pharmaceutical companies that are willing to assume local regulatory and commercial responsibilities could facilitate Japan’s participation in pivotal MRCTs and may ultimately contribute to pediatric approvals in Japan. Third, when direct pediatric trials or early global participation are unfeasible, effective extrapolation and alternative evidence generation become critical, requiring refined regulatory frameworks and enhanced expertise across industry and regulatory authorities. The adoption of advanced complementary approaches such as Modeling and Simulation (M&S), Model-Informed Drug Development (MIDD), and Bayesian information borrowing should be promoted to leverage existing data and reduce the pediatric development burden. These approaches align with ICH E11 (R1), which emphasizes efficient use of existing data, including extrapolation, in pediatric development (International Council for Harmonisation ICH, 2017). In addition, recent FDA guidance highlights the increasing importance of Bayesian frameworks in regulatory decision-making (U.S. Food and Drug Administration, 2026b). Finally, drug development failure is not driven by isolated factors but reflects structural dynamics centered on development stage. Achieving global synchronization through coordinated efforts among regulatory authorities, industry, and academia may help ensure equitable access to pediatric oncology treatments.

This study has several limitations. First, Japanese pediatric approval status was defined based on approved labeling information; therefore, some drug-indication pairs currently under regulatory review or development in Japan may have been misclassified as apparent drug loss despite the possibility of future approval. To reduce this possibility, drug-indication pairs approved in the U.S. after 2023 were excluded from the factor analyses, and an additional sensitivity analysis excluding six ongoing development cases confirmed that our conclusions remained unchanged (Supplementary Tables S3, S4; Supplementary Figures S2, S3); however, residual misclassifications cannot be completely excluded. Second, the number of pediatric oncology drug-indication pairs included in this study was relatively small, reflecting the rarity of pediatric oncology drug development. This limited sample size increases the inherent risk of overfitting in the CART analysis and contributes to wide confidence intervals in the multivariable logistic regression. Although Firth logistic regression and post-pruning of the CART model were applied to address sparse data and mitigate overfitting, respectively, some estimates may still remain unstable. Consequently, the findings from both analyses should be interpreted as exploratory. Third, our evaluation of drug lag using Kaplan-Meier analysis primarily focused on drug-indication pairs that successfully obtained pediatric approval in Japan, with ongoing developments treated as censored. Therefore, the calculated lag time reflects the timeframe for successfully approved therapies.

## 6 Conclusion

This study provides a structural evaluation of the factors underlying pediatric oncology apparent drug loss in Japan. Our findings suggest an evolving dynamic in the Japan-U.S. approval gap: while the pediatric drug lag among successfully approved drug-indication pairs may have shortened to a median of 21.1 months in the post-2017 era, the persistent “not yet approved in Japan”—or “apparent drug loss”—represents a growing challenge that continues to widen disparities in therapeutic access. Our analyses consistently identified the U.S. study phase supporting approval as the factor most strongly associated with Japanese pediatric approval. In particular, the absence of pediatric evidence in the U.S. or development limited to early-phase trials was associated with a structural pattern linked to a higher likelihood of non-approval in Japan. This suggests that because late-phase U.S. evidence is critical for Japanese approval, a drug’s overall likelihood of success may depend on proactively integrating pediatric evaluation during early global development. To mitigate the apparent drug loss and improve access to pediatric oncology therapies, a multi-faceted strategy is essential. First, Japan could strengthen regulatory frameworks that promote early pediatric evaluation based on molecular targets, securing timely inclusion in global trials. Second, industry-academia collaborative mechanisms could help connect U.S. academic-led programs with domestic pharmaceutical companies capable of assuming local regulatory and commercial responsibilities. Finally, for cases where direct global participation is unfeasible, enhancing the practical implementation of extrapolation through advanced methodologies will be important. These strategies may help to address the structural barriers of apparent drug loss and achieve equitable access to innovative therapies for pediatric patients.

## Data Availability

The raw data supporting the conclusions of this article will be made available by the authors, without undue reservation.
